# Antenatal gut microbiome profiles and effect on pregnancy outcome in HIV infected and HIV uninfected women in a resource limited setting

**DOI:** 10.1186/s12866-022-02747-z

**Published:** 2023-01-06

**Authors:** Panashe Chandiwana, Privilege Tendai Munjoma, Arthur John Mazhandu, Jiaqi Li, Isabel Baertschi, Jacqueline Wyss, Sebastian Bruno Ulrich Jordi, Lovemore Ronald Mazengera, Bahtiyar Yilmaz, Benjamin Misselwitz, Kerina Duri

**Affiliations:** 1 Immunology Unit, Department of Laboratory Diagnostic and Investigative Sciences, University of Zimbabwe Faculty of Medicine and Health Sciences, Harare, Zimbabwe; 2 Department of Visceral Surgery and Medicine, Inselspital, Bern University Hospital, University of Bern, 3010 Bern, Switzerland; 3 Department for Biomedical Research, Maurice Müller Laboratories, University of Bern, 3008 Bern, Switzerland; 4 Graduate School for Cellular and Biomedical Sciences, University of Bern, Bern, Switzerland

**Keywords:** HIV infection, Microbiota, Microbiome, Pregnancy, Birth weight

## Abstract

**Background:**

Human immunodeficiency virus (HIV) severely damages the epithelial cells of the gut lining leading to an inflamed leaky gut, translocation of microbial products, and dysbiosis resulting in systemic immune activation. Also, microbiota composition and maternal gut function can be altered in pregnancy through changes in the immune system and intestinal physiology. The aim of this study was to investigate the gut microbiota in HIV-infected and HIV-uninfected pregnant women and to compare and identify the association between gut microbial composition and adverse birth outcomes.

**Results:**

A total of 94 pregnant women (35 HIV-infected and 59 HIV-uninfected controls) were recruited in Harare from 4 polyclinics serving populations with relatively poor socioeconomic status. Women were of a median age of 28 years (interquartile range, IQR: 22.3–32.0) and 55% of women were 35 weeks gestational age at enrolment (median 35.0 weeks, IQR: 32.5–37.2). Microbiota profiling in these participants showed that species richness was significantly lower in the HIV-infected pregnant women compared to their HIV-uninfected peers and significant differences in β-diversity using Bray–Curtis dissimilarity were observed. In contrast, there was no significant difference in α-diversity between immune-compromised (CD4^+^  < 350 cells/µL) and immune-competent HIV-infected women (CD4^+^  ≥ 350 cells/µL) even after stratification by viral load suppression. HIV infection was significantly associated with a reduced abundance of *Clostridium, Turicibacter, Ruminococcus, Parabacteroides, Bacteroides, Bifidobacterium, Treponema, Oscillospira,* and *Faecalibacterium* and a higher abundance of *Actinomyces,* and *Succinivibrio.* Low infant birth weight (< 2500 g) was significantly associated with high abundances of the phylum Spirochaetes, the families *Spirochaeteceae, Veillonellaceae,* and the genus *Treponema.*

**Conclusion:**

The results reported here show that the species richness and taxonomy composition of the gut microbiota is altered in HIV-infected pregnant women, possibly reflecting intestinal dysbiosis. Some of these taxa were also associated with low infant birth weight.

**Supplementary Information:**

The online version contains supplementary material available at 10.1186/s12866-022-02747-z.

## Background

An estimated 35.3 million people worldwide are infected with HIV with more than 2 million new cases since 2012, disproportionately affecting developing countries [1]. About 54% of people living with HIV and acquired immunodeficiency syndrome (AIDS, PLWHA) are from the sub-Saharan Africa (SSA) region [2]. The introduction of combination antiretroviral therapy (cART) has led to a substantial decrease in mortality of PLWHA in the SSA. HIV infection during pregnancy has been a cause of concern in the global goal to mitigate mother-to-child transmission (MTCT). In Southern Africa an HIV prevalence of 16.1% has been recorded, indicating a significant burden of HIV in this population [2–4]. Zimbabwe has an HIV prevalence of 12.8%, with almost 1.4 million PLWHA in 2019 [5]. HIV infection is characterized by progressive loss of cluster of differentiation 4 (CD4^+^) T lymphocytes and chronic systemic immune activation [6].

Some studies have described the occurrence of gut microbial translocations and microbial dysbiosis in HIV-infected individuals, and these processes are regarded as essential pathways for systemic immune activation involving HIV disease progression [7–9]. The causal relationship between altered gut microbiota composition, HIV-induced host immune dysfunction, and cART remains an under-studied area in HIV research [10]. Given that the gut microbiota is closely associated with the host’s immune profile, gut microbial dysbiosis in HIV-infected individuals is a potentially significant contributor to the risk of inflammation-related diseases [11].

HIV infection induces a shift in the gut microbiota composition that is characterized by an enrichment of gram-negative bacteria pathobionts [12–15]. The HIV-related gut dysbiosis is characterized by a decreased abundance of commensal bacteria and an enrichment of potentially pathogenic taxa [16]. Beneficial bacteria such as *Bifidobacterium, Ruminococcus, Bacteroides, Blautia, and Coprococcus* have a lower abundance in HIV infection, whereas *Pseudomonas*, *Enterobacteriaceae, Prevotella, Acinetobacter,* and *Campylobacter,* pathobionts with pro-inflammatory properties, are enriched in HIV infection [6, 16–19].

Pregnancy is a period marked by unique and diverse immune changes which alter the maternal gut function and bacterial composition as the pregnancy advances [20]. The composition of the maternal gut microbiome contributes to obstetric outcomes with long-term health sequelae for mother and child [21]. A healthy pregnancy is usually characterized by an increase in the bacterial load and changes in the composition of gut microbiota [22, 23]. Pregnancy is characterized by a reduction in individual richness (α-diversity) and an increased abundance of the phyla Actinobacteria, Proteobacteria, and the genus *Faecalibacterium* [20, 23, 24]. In other reports, the abundance of the phylum Firmicutes and the genera *Lactobacillus* and *Bacteroides* have been reported to be increased in pregnancy [20, 25, 26].

There is increasing evidence suggesting that the gut microbiome plays a role in HIV transmission and pathogenesis [27, 28]. Understanding the interplay between the gut microbiome and HIV in pregnancy is thus of great value for developing alternative, effective and safer strategies for HIV management such as the use of prebiotics and probiotics. However, most studies on the gut microbiota in patients with HIV were performed in Western populations [6, 27, 29, 30] and the genetics, ethnic background, environment, dietary habits, and lifestyles of these populations differ from African populations. The aim of our study was to investigate the gut microbiota in HIV-infected pregnant women in the third trimester, assess microbiota-related adverse birth outcomes, and determine risk factors associated with gut microbial composition.

## Results

### Characteristics of the study population

Of 97 pregnant women, a total of 94 (35 HIV-infected and 59 HIV-uninfected controls) with available stool samples were included in this study. Sociodemographic and clinical characteristics were compared between HIV-infected pregnant women and HIV-uninfected controls in Table 1. HIV-infected women were significantly older and more likely to have higher number of past pregnancies. There were no significant differences in reported household food security, number of meals eaten per day and not eating enough meals because there was not enough food. However, we note that money spent on food was significantly higher in the HIV-infected individuals. There were no significant differences in marital status, level of education, or religion.

**Table 1:** Clinical and socio-demographic parameters of study participants and infants stratified by HIV status. Data are expressed as n (%) or median (interquartile range, IQR)

**Variables**	**HIV status; *N* = 94**		***p*-value**
**Maternal HIV status**	**Positive: *N* = 35**	**Negative: *N* = 59**	
	**Median (IQR)**	**Median (IQR)**	
**Sociodemographic descriptors**			
**Maternal age (years)**	32.0 (28.0–34.5)	24.0 (21.0–29.0)	**<0.0001**
**Gestational age (weeks) at enrolment**	34.3 (32.7–36.7)	35.5 (32.5–37.3)	0.718
**Number gravida**	3.0 (3.0–4.0)	2.0 (1.0–3.0)	**<0.0001**
**Religion**			
Apostolic	12 (36%)	27 (79%)	0.263
Non-apostolic	21 (64%)	25 (21%)	
	3 missing	9 missing	
**Marital status**			
Not married	2 (6%)	1 (2%)	0.553
Married	34 (94%)	60 (98%)	
**Alcohol use in pregnancy**			
Yes	2 (6%)	6 (10%)	0.706
No	34 (94%)	55 (90%)	
**Meals normally eaten per day**	3.0 (2.0–3.0)	3.0 (3.0–3)	0.349
**Meals eaten during pregnancy**	3.0 (3.0–5.0)	3.0 (2.0–4.5)	0.383
**Fewer meals per days because family can’t afford**			
Yes	5 (14%)	11 (19%)	0.778
Never	30 (86%)	48 (81%)	
**Money spent on food monthly (US Dollars)**	150 (100–200)	100 (70–150)	**0.025**
**Food security**			
Yes	26 (74%)	47 (80%)	0.612
No	9 (36%)	12 (20%)	
**Coinfections and sexually transmitted diseases**			
**Cytomegalovirus (CMV) IgM**			
Positive	2 (6%)	7 (11%)	0.156
Negative	32 (94%)	54 (89%)	
	1 missing		
**Syphilis antibodies**			
Positive	2 (6%)	2 (3%)	0.415
Negative	33 (94%)	59 (97%)	
	1 missing		
**Hepatiris B virus antigen**			
Positive	1 (3%)	1 (2%)	1
Negative	35 (97%)	60 (98%)	
**Reported previous sexually transmitted disease diagnosis**			
Yes	9 (25%)	17 (28%)	0.816
No	27 (75%)	44 (78%)	
**Clinical data**			
**Mid upper arm circumference (cm)**	27.0 (24.8–28.6	24.5 (23.0–27.4)	**0.006**
**Body mass index (kg/m**^**2**^**)**	26.3 (24.5–28.1)	22.7 (21.2–25.8)	**0.001**
**White blood count (10e09/l)**	7.5 (6.0–8.8)	7.6 (6.8–8.7)	0.317
**Platelets (10e09/l)**	235.0 (203.5–265.5)	211.0 (182.0–250.4)	0.586
**Haemoglobin category**			
≤ 11 g/dl	16 (46%)	21 (34%)	0.286
> 11 g/l	19 (54%)	40 (66%)	
	1 missing		
**Antibiotic use**			
Yes	12 (34%)	1 (2%)	**<0.0001**
No	23 (66%)	58 (98%)	
**HIV and cART related factors**			
**Days since HIV diagnosis**	833.5 (115.8–2289.2)		
**Duration of cART use**	820.0 (115.8–1932.2)		
**Ever switched cART regimens**			
Yes	4 (11%)		
No	32 (89%)		
**Cotrimoxazole use**			
Yes	12 (34%)		
No	23 (66%)		
**HIV-RNA (copies per mL)**			
≤ 1000	29 (83%)		
> 1000	6 (17%)		
	1 missing		
**HIV-RNA (copies per mL)**			
< 50	28 (80%)		
> 50–1000	1 (2%)		
> 1000—≤ 10 000	2 (6%)		
> 10,000	4 (11%)		
	1 missing		
**CD4 cells/µL**			
< 350	15 (44%)		
≥ 350	19 (56%)		
	2 missing		
**Pregnancy outcomes (*N* = 95 infants, including 1 sets of twins)**			
**Delivery < 37 weeks gestational age**			
Yes	3 (9%)	8 (14%)	0.741
No	32 (91%)	50 (86%)	
	1 missing	1 missing	
**Birth status**			
Alive birth	37 (100%)	59 (100%)	1
Still births	0	0	
**Birth weight category**			
≤ 2500	4 (11%)	8 (14%)	1
> 2500	31 (89%)	51 (86%)	
	1 missing		
**Head circumference (cm)**	34.0 (34.0–35.8)	34.0 (33.0–35.0)	0.068
**Birth length (cm)**	50.0 (49.0–51.0)	49.0 (48.0–50.3)	0.586

HIV-infected women had a significantly higher mid-upper arm circumference  (MUAC) and body mass index (BMI). Sexually transmitted diseases (STD), including cytomegalovirus (CMV), syphilis, and hepatitis B virus (HBV) did not differ between HIV-infected and HIV-uninfected women. Antibiotic use was significantly higher in the HIV-infected compared to HIV-uninfected controls and the majority of the antibiotic use in the HIV-infected could be attributed to cotrimoxazole (34%). Eighty-three percent of the HIV-infected individuals had a viral load lower than 1000 copies/mL and 44% had a CD4^+^ T-lymphocyte count lower than 350 cells/µL.

### HIV infection and the intestinal microbiome

#### Species richness and diversity in HIV infected mothers

**Fig. 1:**
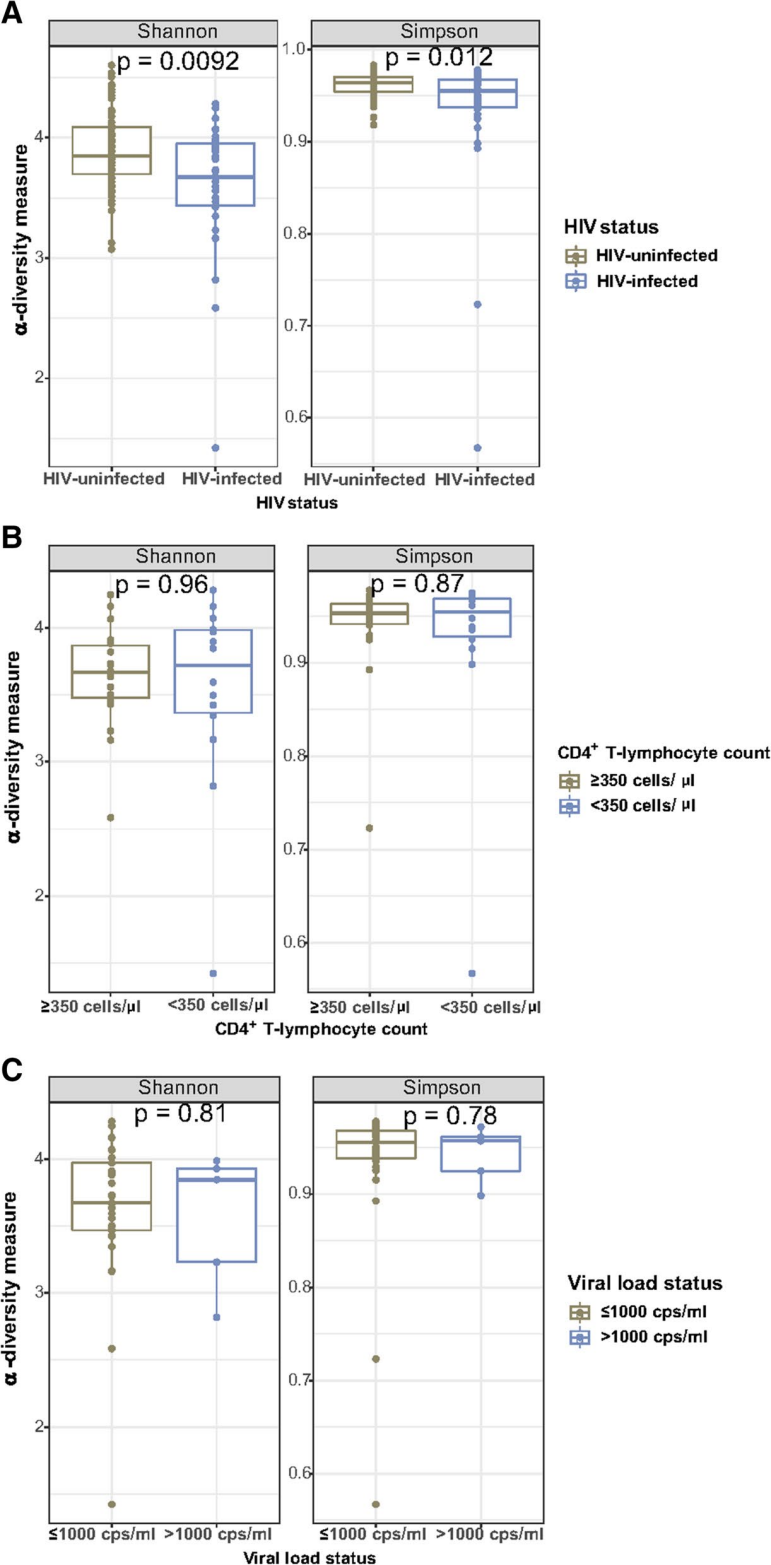
Differences in species α-diversity of the gut microbiota in pregnancy at least 20 weeks gestational age. Comparisons between (**A**) HIV-infected and HIV-uninfected controls, (**B**) HIV-infected women stratified by immune status (CD4^+^ T-lymphocyte count) and (**C**) HIV-infected women stratified by viral load are shown. Statistics: Mann–Whitney U test

Bacterial community α-diversity measures consistently showed a lower α-diversity in the HIV-infected women than in the HIV-uninfected controls according to the Shannon index (*p* = 0.0092) and Simpson’s index (*p* = 0.012), respectively (Fig. 1A). When stratified by CD4^+^ T-lymphocyte counts, no significant differences between the immune-compromised group (CD4^+^  < 350 cell/µL) and the immune-competent group (CD4^+^  ≥ 350 cell/µL) group was detected for α-diversity (Fig. 1B). HIV virally suppressed pregnant women defined by VL ≤ 1000 cps/ml did not display a significantly different bacterial α-diversity compared to unsuppressed pregnant women (VL > 1000 cps/ml) (Fig. 1C).

**Fig. 2:**
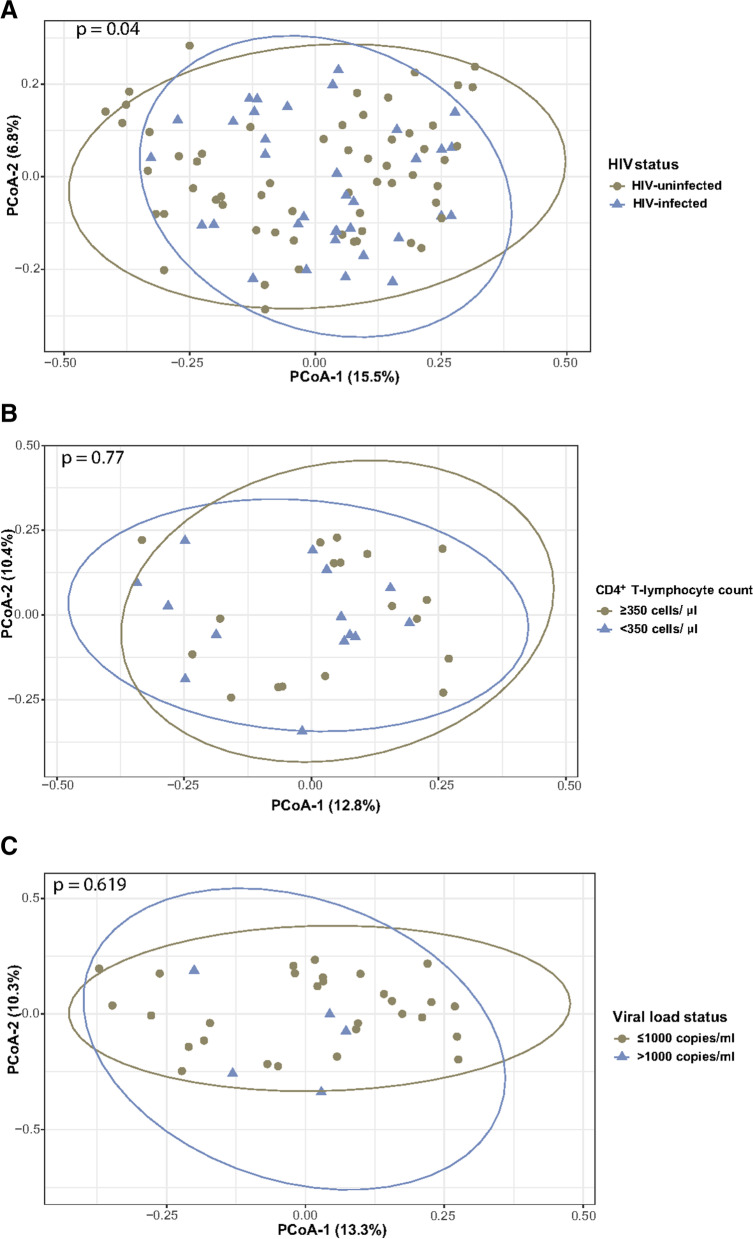
Characterization of β-diversity of the gut microbiota in pregnancy at least 20 weeks gestational age. Groups stratified by (**A**) HIV status (**B**) immune status (CD4^+^ T-lymphocyte count) (**C**) viral load status were analyzed by Bray–Curtis dissimilarity. Each dot represents a single fecal sample. Statistics: PERMANOVA

PCoA plots of Bray–Curtis dissimilarity of HIV-infected and uninfected women largely overlapped (Fig. 2A), although a significant association of β-diversity with HIV status was observed in the PERMANOVA analysis (*p* = 0.04). Upon stratification according to CD4^+^ T-lymphocyte counts, there was no significant difference in β-diversity between the immune compromised (CD4^+^  < 350 cell/µL) and the immune competent (CD4^+^  ≥ 350 cell/µL) groups in the PERMANOVA analysis (*p* = 0.77) (Fig. 2B). There was also no significant difference in β-diversity between the HIV suppressed (VL ≤ 1000 cps/ml) and unsuppressed (VL > 1000 cps/ml) pregnant women (Fig. 2C).

### Association of microbiota taxa with HIV infection status

**Fig. 3:**
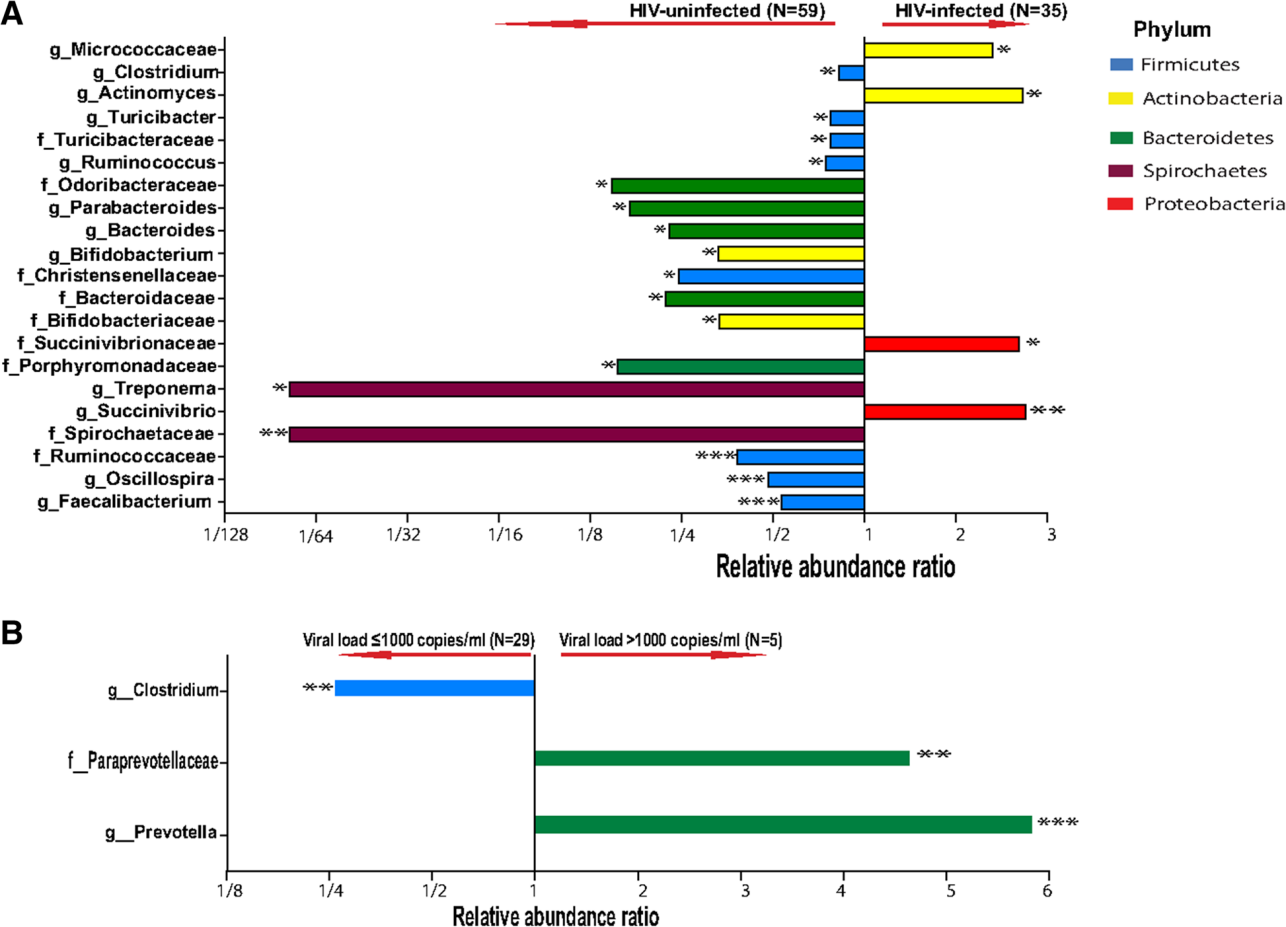
Microbiota abundance according to HIV infection status in pregnant women at least 20 weeks gestational age. Taxonomic differences between the gut microbiota of (**A**) HIV-infected women and healthy controls and (**B**) groups stratified by viral load were compared by MaAsLin2 analysis. *: *p* value < 0.05, **: *p* value < 0.01, ***: *p* value < 0.001

HIV-infected individuals and HIV-uninfected controls have similar taxonomy profiles and the majority of the most abundant bacterial OTUs were identical between both groups (Supplementary Fig. 1A and B). Statistical taxonomy analysis using MaAsLin2 indicated that HIV infection was significantly associated with a lower abundance of the families *Turibacteraceae, Odoribacteraceae, Christensenellaceae, Bacteroidaceae, Bifidobacteraceae, Porphyromonadaceae, Spirochaetaceae, Ruminococcaceae* and at genus level of *Clostridium, Turicibacter, Ruminococcus, Parabecteroides, Bacteroides, Bifidobacterium, Treponema, Oscillospira* and *Faecalibacterium*. HIV infection was also associated with a higher abundance of *Micrococcaceae* and *Succinivibrionaceae* at the family level and the genera *Actinomyces,* and *Succinivibrio* (Fig. 3A). *Ruminococcaceae, Oscillospira* and *Faecalibacterium* remained significantly lower in HIV infected women after correcting for false discovery (q < 0.05).

### Associations of microbiota taxa with HIV infection parameters

When we investigated the association of CD4^+^ T lymphocyte counts and the gut microbiota, we did not find any significant associations with an immune-compromised state (CD4^+^  < 350 cells/µL) and an immune-competent state (CD4^+^  ≥ 350 cells/µL, data not shown). We also investigated the association of gut microbiota with viral load (VL) using suppression cutoffs as per WHO guidelines (VL ≤ 1000 cps/ml vs. VL > 1000 cps/ml) (Fig. 3B). Higher abundance of genus *Clostridium* was significantly associated with a VL < 1000 cps/ml whilst higher abundances of family *Paraprevotellaceae* and genus *Prevotella* was associated with a VL > 1000 cps/ml and only *Prevotella* remained significant after false discovery rate correction (q < 0.05). We did not find any significant associations between parameters of the intestinal microbiota and duration of cART use.

### Microbiota and birth outcomes

**Fig. 4:**
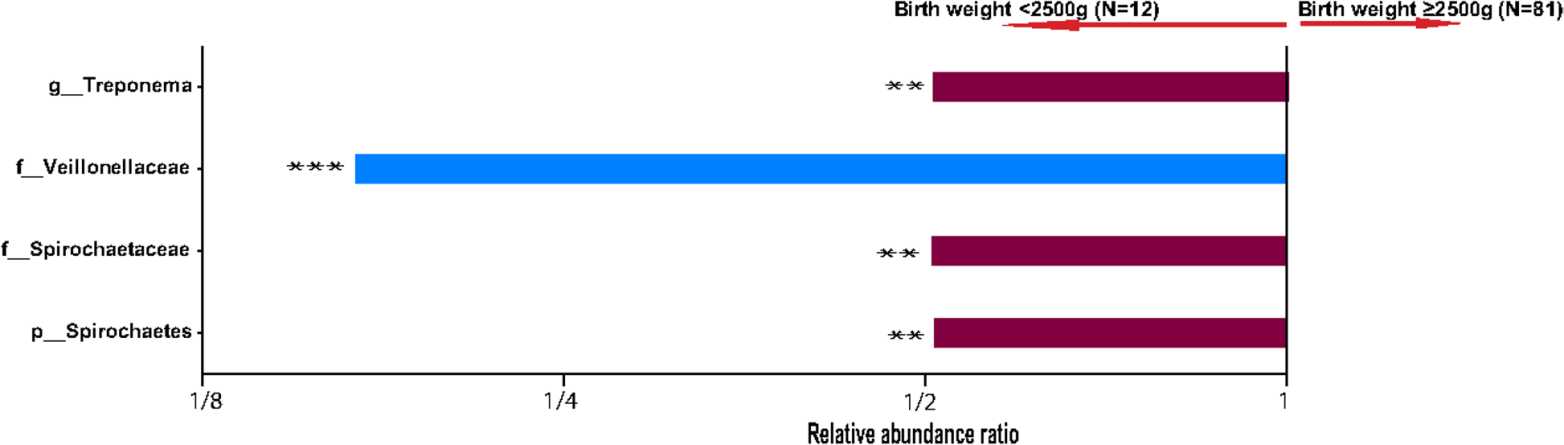
Microbiota abundance and birth outcome. Taxonomic differences between the gut microbiota of groups stratified by baby birth weight were compared by MaAsLin2 analysis. *: *p* value < 0.05, **: *p* value < 0.01 = **, ***: *p* value < 0.001

We investigated the effects of the gut microbiota in pregnancy on pregnancy and birth outcomes. Low infant birth weight (< 2500 g) was significantly associated with high abundances of the phyla Spirochaetes, the families *Spirochaeteceae,* and *Veillonellaceae* and the genus *Treponema* (Fig. 4) though these associations lost significance after false discovery rate correction (q > 0.05). No significant associations were observed between the gut microbiota and infant birth head circumference as well as pregnancy term at birth.

## Discussion

In this study we assessed the composition of the intestinal microbiota in pregnant women residing in a low-resource setting, facing significant economic and public health challenges. HIV-infected pregnant women had a lower α-diversity than controls with β-diversity also differing significantly between cases and controls. The gut bacterial communities in HIV-infected pregnant women showed an enrichment in the taxa *Micrococcaceae, Succinivibrionaceae*, *Actinomyces,* and *Succinivibrio.* High abundance of the taxa Spirochaetes, *Spirochaeteceae, Veillonellaceae* and *Treponema* in the third trimester of pregnancy was associated with low birth weight in infants.

The lower α-diversity of the gut microbiota of HIV-infected pregnant women in our study concurs with other findings that have reported a reduced α-diversity in the gut microbiota during HIV infection [9, 29, 31, 32]. The lower diversity might be due to an HIV-related deterioration of the gut-associated lymphoid tissue, leading to dysbiosis and microbial translocation. Thus, while our data confirm the results from other studies [9, 29, 31, 32], it should be noted that most of these studies were carried out in non-pregnant women, and none were from the SSA region.

When we further stratified the HIV-infected group by immune competence, we observed no differences in the α- diversity between the immune-compromised (CD4^+^  < 350 cells/µL) and the immune-competent (CD4^+^  ≥ 350 cells/µL) groups. These findings contrast previous studies which observed a significantly lower α-diversity in the immune-compromised group, linking reduced gut microbiota diversity to immune dysfunction and reduced CD4^+^ T-lymphocyte counts [8, 29]. This discrepancy might be due to the larger study population in the two previous studies which also investigated a mixed population of males and females, compared to our study with a study population of pregnant women. In our study, we also did not find a significant difference between the HIV viral suppressed (VL ≤ 1000 cells/ml) and the viral unsuppressed group (VL > 1000 cells/ml), in line with a Zimbabwean study investigating a different population [33].

There was a trend for a lower α-diversity with a longer duration on cART. Some studies found a negative impact of cART on gut microbiota diversity [29, 34]. For instance, Nowak et al. observed a decrease in α-diversity after the initiation of cART for the next 10 months. In our study we did not investigate the effect of cART longitudinally which might have limited our capability to assess the actual effect of cART.

Published data are less consistent regarding the relative abundance of specific taxa in HIV-infected individuals [6, 9, 19, 29, 31, 32, 35]. Types of specimens used, study populations, geographical area, sequencing method, and false discovery may explain some of the differences identified.

In our study, HIV infection was associated with a lower abundance of the *Ruminococcaceae* family, *Clostridium, Ruminococcus, Faecalibacterium,* and *Oscillospira*. All of these four taxa belong to the Clostridia class, which have been reported to be particularly impacted by HIV infection [36]. *Faecalibacterium* with known anti-inflammatory properties [37] has been reported to be significantly reduced in HIV infection [38], similar to our findings. The depletion of the *Clostridium* genus has been highlighted [29], and has been reported to attenuate inflammation and allergic diseases effectively owing to their distinctive biological activities [39]. A clear role for these bacterial families in health and disease has not been well established yet. A function of the genus *Ruminococcus* in the glycerophospholipid metabolism pathway has been suggested, which correlates with elevated plasma glycerophospholipid levels in women with chronic HIV infection [40]. We found a low abundance of the genus *Oscillospira* in the HIV-infected group which was contrary to the findings by Wang et al. [40] who observed enrichment in HIV-infected non-pregnant participants.

We found a reduced abundance of two potentially beneficial genera, *Bacteroides* and *Bifidobacterium,* in the HIV-infected group. *Bacteroides* has frequently been reported to be depleted in HIV-infected subjects [15, 41–43]. This taxa has anti-inflammatory properties and its reduction might be responsible for the maintenance of systematic inflammation usually observed in HIV infection [13]. The low abundance of *Bifidobacterium* in HIV-infected women in this study is supported by findings in previous studies in non-pregnant participants [40, 44]. *Bifidobacterium*, a genus of anti-inflammatory short-chain fatty acids (SCFA) producing bacteria [45, 46] has probiotic properties in the human gut [40] and its reduction in the HIV-infected group might be of interest as SCFA-producing bacteria have been speculated to play a key role in neuro-immunoendocrine regulation [47]. Taken together, the depletion of two potentially beneficial microbiota genera, the *Bacteroides* and *Bifidobacterium* are in line with the adverse effects of HIV on microbiota health.

In the current study, HIV infection was associated with a higher abundance of the genus *Succinivibrio*. The enrichment of *Succinivibrio* has been reported in HIV-infected people undergoing cART treatment [48]. *Succinivibrio* increases are associated with defects in gastrointestinal functions, such as diarrhea and abdominal pain [49, 50]. Since all HIV-infected participants in our study were on cART, cART might either cause gastrointestinal side effects, supporting the enrichment of *Succinivibrio* and/ or directly impact on *Succinivibrio*, which could mediate gastrointestinal symptoms [48, 49]*.* In any case, a potential association of cART and *Succinivibrio* could be a useful therapeutic target for limiting the side effects of cART and should be investigated further.

We also observed an association of low abundance of the phylum Spirochaetes, and the families *Spirochaetaceae*, and *Veillonellaceae*, and the genus *Treponema* with low infant birth weight. In contrast to our study, Gough et al*.* found that taxa associated with resistant starch degradation, specifically members of the *Ruminococcaceae, Lachnospiraceae*, and *Eubacteriaceae* families, were the most important predictors of birth weight [51].

Strengths of this study include (i) that all our participants were recruited in the same low resource setting with homogenous environmental exposure and (ii) the participants were clinically and socio-economically characterized at a high level of detail. Limitations of our study include the non-interventional study design, making disentangling of the causal relationship between gut microbiota, HIV infection, immune dysfunction and HIV treatment difficult. Also, for this study, no longitudinal samples were analyzed and our sample size is small, which limits our analysis especially when subgroups of HIV-infected women are assessed.

## Conclusion

Our study is among the first to assess the gut microbial composition of HIV-infected pregnant women in a low-resource setting with a high burden of HIV. The results show that the diversity and composition of the gut microbiota are changed in HIV-infected pregnant women and are potentially associated with microbial alterations. Normalization of microbiota might therefore be a relevant therapeutic outcome for future longitudinal HIV studies and large prospective studies of the intestinal microbiota and HIV infection, markers of microbial translocation, immunological markers and the host’s immune response are warranted.

## Methods and materials

### Study population

This is a cross-sectional sub-study nested in the University of Zimbabwe Birth Cohort Study (UZBCS). The methodology of the UZBCS has been described elsewhere [52], in brief: Study participants were recruited during routine antenatal care visits at four City of Harare Polyclinics namely, Kuwadzana, Rujeko, Budiriro, and Glenview, serving populations with relatively poor socio-economic status. The target population of the study comprised pregnant women of at least 20 weeks` gestational age with plans to deliver at one of the four study sites and who were willing to consent for themselves and give parental consent for their child’s participation. Women below the age of 15 years and/ or with severe mental health disorders, compromising their ability to provide informed consent, were excluded from the study. Clinical, sociodemographic, and nutritional information was collected using pretested and approved questionnaires. A physical examination was carried out which included measurements of height, pregnancy weight, MUAC, and BMI. Written informed consent was obtained from all participants and parental consent for their infants to participate in the study. Ethical clearance for the study was obtained from the Joint Research Ethics Committee (JREC/228/19) and the Medical Research Council of Zimbabwe (MRCZ/B/1824). UZBCS has been registered at www.clinicaltrials.gov with identifier NCT04087239 on 12/09/2019.

This study focuses on a subset of all UZBCS participants, enrolled from February 2019 to September 2019. A total of 97 pregnant women (36 HIV-infected and 61 HIV-uninfected controls) were thus included.

### Laboratory assays for the determination of CD4+ T-lymphocyte count, HIV viral load, full blood count, and plasma biomarkers

Plasma was isolated from whole blood within 6 h of sample collection and stored at -80 °C. HIV was diagnosed using Determine HIV*-*1/2 kit (Abbott Diagnostics, Chiba, Japan) a qualitative rapid immuno-chromatographic assay*.* CD4^+^ T-lymphocyte counts in ethylenediaminetetraacetic acid (EDTA) blood samples were enumerated within a maximum of 6 h after sample acquisition for all HIV-infected women using a Partec Cyflow counter (Cyflow, Partec, Munster, Germany). Full blood counts (FBC) were determined from whole blood samples using a Mindray Haematology 3-part differential, BC3600 Analyser (Shengzhen, China). HIV RNA was extracted from 1 ml maternal frozen plasma and quantified using an automated Nuclisens EasyQ bioMérieux Clinical Diagnostics, Marcy-l'Étoile, France. The detection limit for the assay is 100 cps/ml.

For HIV [8] viral load categorization, we used the World Health Organization (WHO) guidelines with a cut-off of ≤ 1000 cps/ml for viral suppression and a viral load (VL) > 1000 cps/ml indicating an unsuppressed viral load. We also used WHO guidelines to group by immune competence using CD4^+^  < 350 cells/µL for immune incompetence and CD4^+^  ≥ 350 cells/µL for the immune-competent group [53]. Cutoffs for cART duration were used as described by Duri et al*.* in the same cohort [54].

### Extraction of genomic DNA and microbial profiling

Fresh fecal samples were collected in a sterile plastic cup and transferred immediately to the laboratory and stored at -80℃. The samples were aliquoted, and bacterial DNA extraction was carried out using a Qiagen Power Fecal Pro kit (Qiagen, Dusseldorf, Germany) according to the manufacturer’s instructions and specifications. The DNA samples were stored at -20 °C prior to the polymerase chain reaction (PCR) assay.

The 16S rRNA gene segments spanning the variable V5 and V6 regions were amplified with Invitrogen Platinum Taq DNA polymerase from DNA using a range of oligonucleotide primers with specificity for the rDNA bacteria domains V5 and V6. All forward core primers were specifically modified by the addition of a Personal Genome Machine (PGM) sequencing adaptor, a ‘GT’ spacer and a unique barcode that allowed to have up to 96 unique barcodes. The expected product length was ~ 350 base pairs including adaptors and barcodes. Barcoded forward primer 5′-*CCATCTCATCCCTGCGTGTCTCCGACTCAG* BARCODE ATTAGATACCCYGGTAGTCC-3′ in combination with the reverse fusion primer 5′-*CCTCTCTATGGGCAGTCGGTGAT*ACG AGCTGACGACARCCATG-3′ were used; sequences in *italics* indicate Ion torrent PGM-specific adapter sequences [55, 56]. PCR conditions consisted of an initial 5 min denaturation step at 94 °C, followed by 35 cycles of 1 min denaturation at 94 °C, a 20 s annealing cycle at 46 °C, and a 30 s extension cycle at 72 °C, with a final extension for 7 min at 72 °C.

The PCR product was kept at 4 °C until loading into a 1% agarose gel and run on gel electrophoresis for 1 h. The PCR amplicon was purified from the gel using Gel Extraction Kit (Qiagen). The concentrations of the PCR amplicon was then evaluated using a Qubit 3.0 Fluorometer (ThermoFisher) prior to library preparation. To prepare template-positive Ion PGM Template OT2 400 Ion Sphere Particles containing clonally amplified DNA, we used the Ion OneTouch Instrument with the Ion PGM Template OT2 400 Kit (ThermoFisher). Sequencing was performed using the Ion PGM Sequencing 400 Kit and Ion 316 Chip V2 within the Ion PGM System (ThermoFisher) [55, 57].

### Statistical analysis

Characteristics of pregnant women with and without HIV diagnosis were compared using the Mann–Whitney U test (continuous variables) and Fisher’s Exact test (categorical variables), respectively. Reads were demultiplexed and preprocessed using the *q2-cutadapt* plugin after importing multiplexed FASTQ artifacts into Quantitative Insights into Microbial Ecology 2 (QIIME2) [58] version 2021.11.0. The sequence barcodes were trimmed off using q2- *cutadapt trim* to remain with target sequences. A summary of demultiplexed artifacts was created using *q2-demux summarize.* Sequence quality control and feature table construction were performed using denoiser *q2-dada2* [59] which corrects amplicon sequence errors and produces high-resolution amplicon sequence variants (ASVs). The feature table is a table of the counts of each observed feature in each sample and is used to generate a phylogenetic tree. The feature table was filtered so that it only contained fragments that were in the insertion tree. Taxonomic classification was carried out using the *q2-feature-classifier* plugin and a Naive Bayes classifier, which is trained on taxonomically defined reference sequences covering the target region of interest. Taxonomic weights were assembled with16S rRNA gene sequence data using the SILVA reference database [60].

The feature table and mapping file were used for statistical analyses and data visualization in the R statistical programming (version 4.1.2) environment package *phyloseq* [61]. Alpha diversity was calculated using the Shannon and Simpson’s diversity indices. Mann–Whitney U tests were applied to compare the differences in the microbial α-diversity indices (Shannon index and Simpson index) by HIV status. β-diversity was calculated by Bray–Curtis genus level community dissimilarities based principal coordinate analysis (PCoA). Statistical analysis by permutational multivariate analysis of variance (PERMANOVA) was used to statistically analyze the microbial β-diversity and confirm the strength and statistical significance of group association in the same distance metrics [62]. Taxonomy analyses were performed with the MaASlin2 package [63] in R statistical programming. Plots were generated using phyloseq object or GraphPad Prism v7.

## Supplementary Information

**Additional file 1: Supplementary Figure 1.** Abundance plots of the gut microbiota in pregnancy at least 20 weeks gestational age stratified by HIV status. (A) abundance plot at phylum level (B) abundance plot at genus level.

## Data Availability

The datasets used and analysed during the current study are available from the corresponding author upon request.

## Abbreviations

AIDS: Acquired immunodeficiency syndrome
BMI: Body mass index
CMV: Cytomegalovirus
cART: Combination antiretroviral therapy
CD4 +: Cluster of differentiation
FBC: Full blood count
HBV: Hepatitis B virus
HIV: Human Immunodeficiency Virus
MTCT: Mother-to-child transmission
MUAC: Mid upper arm circumference
OUT: Operational taxonomic unit
PCoA: Principal coordinate analysis
PLWHA: People living with HIV and AIDS
PERMANOVA: Permutational multivariate analysis of variance
QIIME2: Quantitative Insights into Microbial Ecology 2
STD: Sexually transmitted diseases
SCFA: Short-chain fatty acids
SSA: Sub-Saharan African
UZBCS: University of Zimbabwe Birth Cohort Study
VL: Viral load
WHO: World Health Organisation
