# Supplementary material for: Antenatal gut microbiome profiles and effect on pregnancy outcome in HIV infected and HIV uninfected women in a resource limited setting
Source: BMC Microbiol. 2023 Jan 6;23:4. doi: 10.1186/s12866-022-02747-z (PMC9817306; doi:10.1186/s12866-022-02747-z)
Supplement: Supplementary file 1 — Additional file 1: Supplementary Figure 1. Abundance plots of the gut microbiota in pregnancy at least 20 weeks gestational age stratified by HIV status. (A) abundance plot at phylum level (B) abundance plot at genus level. [file 12866_2022_2747_MOESM1_ESM.pdf]

Supplementary Figure 1A

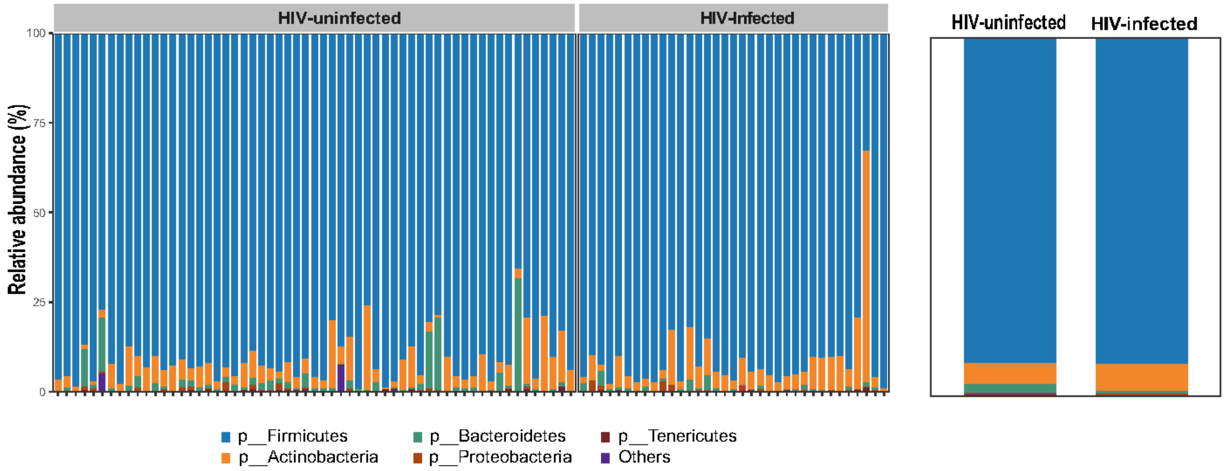

Supplementary Figure 1B

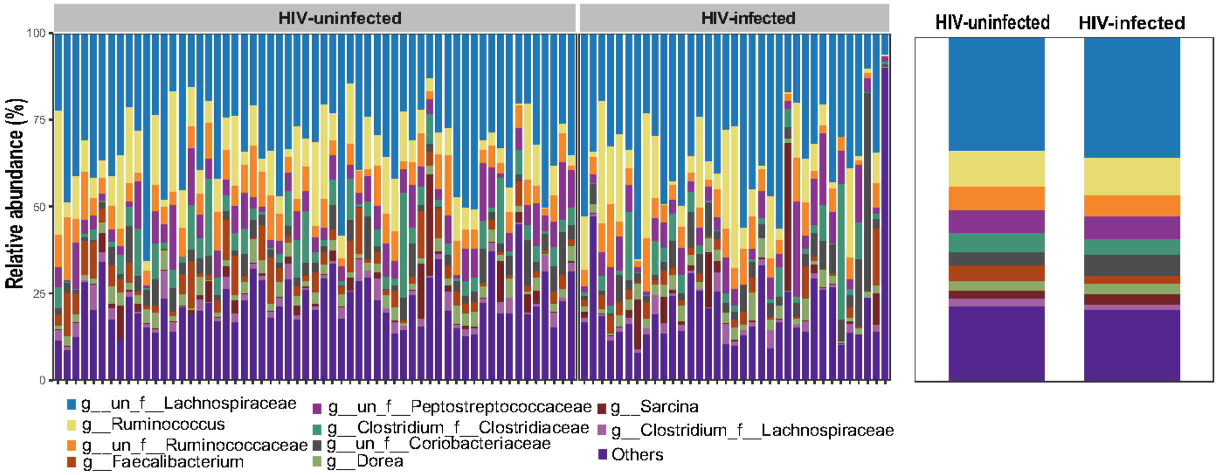

Supplementary Figure 1. Abundance plots of the gut microbiota in pregnancy at least 20 weeks gestational age stratified by HIV status. (A) abundance plot at phylum level (B) abundance plot at genus level.
